# A Pilot Randomized Control Trial Testing a Smartphone-Delivered Food Attention Retraining Program in Adolescent Girls with Overweight or Obesity

**DOI:** 10.3390/nu16203456

**Published:** 2024-10-12

**Authors:** Megan N. Parker, Bess F. Bloomer, Jeffrey D. Stout, Meghan E. Byrne, Natasha A. Schvey, Sheila M. Brady, Kong Y. Chen, Allison C. Nugent, Sara A. Turner, Shanna B. Yang, Monika M. Stojek, Andrew J. Waters, Marian Tanofsky-Kraff, Jack A. Yanovski

**Affiliations:** 1 Section on Growth and Obesity, Division of Intramural Research, *Eunice Kennedy Shriver* National Institute of Child Health and Human Development (NICHD), National Institutes of Health (NIH), Department of Health and Human Services (DHHS), 10 Center Drive, Room 1-3330, Bethesda, MD 20892, USA; megan.parker.ctr@usuhs.edu (M.N.P.); bessfbloomer@gmail.com (B.F.B.); natasha.schvey@usuhs.edu (N.A.S.); bradys@mail.nih.gov (S.M.B.); 2 Department of Medical and Clinical Psychology, Uniformed Services University of the Health Sciences (USUHS), 4301 Jones Bridge Road, Bethesda, MD 20814, USA; andrew.waters@usuhs.edu; 3 MEG Core Facility, National Institute of Mental Health (NIMH), National Institutes of Health (NIH), Department of Health and Human Services (DHHS), Bethesda, MD 20892, USA; jeff.stout@nih.gov (J.D.S.); nugenta@mail.nih.gov (A.C.N.); 4 Section on Development and Affective Neuroscience, Emotion and Development Branch, National Institute of Mental Health (NIMH), National Institutes of Health (NIH), Department of Health and Human Services (DHHS), Bethesda, MD 20892, USA; meghan.byrne2@nih.gov; 5 Diabetes, Endocrinology, and Obesity Branch, Division of Intramural Research, National Institute of Diabetes and Digestive and Kidney Diseases, National Institutes of Health (NIH), Department of Health and Human Services (DHHS), Bethesda, MD 20892, USA; chenkong@niddk.nih.gov; 6 Nutrition Department, Clinical Center, National Institutes of Health (NIH), Department of Health and Human Services (DHHS), 10 Center Drive, Bethesda, MD 20892, USA; sara.turner@nih.gov (S.A.T.); shanna.bernstein@nih.gov (S.B.Y.); 7 Institute of Psychology, University of Silesia in Katowice, 40-007 Katowice, Poland

**Keywords:** attention bias, obesity, adolescence, attention retraining, smartphone program

## Abstract

Background/Objectives: Attention bias (AB) toward food is associated with obesity, but it is unclear if programs designed to reduce AB can impact adolescents’ eating behavior. We investigated whether a two-week, smartphone-delivered attention retraining (AR) program (vs a control program) altered food AB in adolescent girls with overweight. Methods: Participants completed three food-cue visual-probe trainings/day. The AR and control programs directed attention away from food stimuli during 100% and 50% of trainings, respectively. Before and after completion of the programs, girls completed a food-cue visual-probe task while undergoing magnetoencephalography (MEG), and then a laboratory test meal. Results: Sixty-eight adolescents were randomized; 58 completed post-program visits. There was minimal effect of condition on AB scores (β [95%CI] = −1.9 [−20.8, 16.9]; *d* = −0.06). There was a small effect of condition on energy intake (EMM_control_ = 1017 kcal, EMM_AR_ = 1088 kcal, *d* = 0.29). Within the AR group, there was slightly blunted initial engagement in brain areas associated with reward response and subsequent increased goal-directed attention and action control. Conclusions: We found preliminary support for efficacy of an intensive smartphone-delivered AR program to alter neural correlates of attention processing in adolescent girls with overweight or obesity. Studies with larger sample sizes are needed to elucidate if AR trainings disrupt the link between food AB and eating behavior.

## 1. Introduction

Food attention bias may be a useful target for interventions aiming to reduce aberrant eating and slow unhealthy weight gain during adolescence. Attentional bias (AB) is a tendency to attend selectively to environmental stimuli, such as food, that have acquired salience or meaning [1,2]. Although food AB is not inherently problematic, several reviews support an association between increased food AB and aberrant eating behavior [3,4,5] and obesity [6,7,8]. While there is some evidence that children with and without overweight do not differ in their attention towards food [9,10], other reports indicate a positive cross-sectional association between food AB and body mass index (BMI, kg/m^2^) in children and adolescents [11,12]. Moreover, food AB has been shown to prospectively predict weight gain in adolescents [11]. Food AB might promote weight gain due to its association with aberrant eating behavior, such as increased energy intake [4,13] and loss-of-control eating [3] (LOC-eating; the subjective experience of a lack of control over what or how much is eaten [14]). One study found that the link between food AB and weight was significant only among adolescents with recent experiences of LOC-eating [15]. Similar positive associations between AB and snack food intake [16], LOC-eating [3,17,18], and overweight and obesity (compared to average weight) [16,19,20] have been reported among adults. Therefore, adolescence might be an opportune period for disrupting the development of associations between food AB and obesity.

Assessments of neural activity have been a key part of attempts to understand food AB [6,11,21]. Evidence that weight is associated with food AB is stronger when AB processes are assessed by neuroimaging (e.g., electroencephalogram, functional magnetic resonance imaging), and weaker or mixed when assessed by behavioral tasks [4,8,22]. The neural processes involved in attention are deployed over short time scales (milliseconds), and are therefore optimally captured by electroencephalogram and magnetoencephalography, temporally sensitive neuroimaging methods [23]. Specifically, rapidly deployed “bottom-up” striatal circuitry is involved in unconscious attention deployment, as well as food cue processing and reward value encoding [24,25,26,27,28,29,30]. Unconscious attention deployment during food AB tasks has also been linked to hyperactivation in the insula, ventral anterior cingulate cortex (ACC), and orbitofrontal cortex (OFC) in adolescent girls [11] and adults with LOC-eating [21,31,32]. Conscious attention deployment, involving “top-down” brain regions that support task-related goal attainment including the dorsal ACC and the ventrolateral and dorsolateral prefrontal cortex (vlPFC, dlPFC, respectively; [2]), occurs after unconscious attention deployment. Thus, there are two interacting pathways through which interventions aiming to reduce AB might affect change [33]; by affecting unconscious attention deployment, e.g., by reducing the reward responsivity (Valence-Specific Models), or altering conscious attention deployment, e.g., by increasing attentional control (Attentional Control Models).

Attention retraining (AR) programs, also commonly referred to as attention bias modification programs, are designed to alter attention processing. Extant AR programs have demonstrated preliminary efficacy for reducing food AB and energy intake among adults [34,35,36]. A recent review of ten AR programs delivered in a laboratory setting found that the trainings acutely reduced food AB [37]. There is also preliminary evidence that AR programs evince changes in reward and attention related neural underpinnings of food AB, suggesting these interventions might exert their effect by reducing the reward valuation of food [38]. However, there are limited data demonstrating that food-based AR programs affect changes in eating behavior and weight [37,39], potentially due to a lack of understanding as to how each phase of attention (capture and deployment) relates to behavioral outcomes [4].

Additionally, the dose of extant food-based AR programs, which range from a single training to 30 trainings delivered over the course of 5 weeks [37], may be too low to impact eating behavior. Higher intensity AR programs that require the completion of several AR trainings delivered multiple times a day for multiple weeks may increase the effectiveness of AR programs [40,41,42,43]. The use of smartphones for intervention delivery makes more intensive AR programs feasible, especially for adolescents. Smartphones have become an integral part of society, with rates of smartphone ownership among youth dramatically increasing in the last decade [44,45]. Compared to laboratory/clinic-based interventions, programs delivered on a smartphone allow for youth to complete more intensive AR programs in their natural environments.

We therefore conducted a double-blind randomized controlled pilot trial to test the impact of a two-week long AR program (compared to a control program) on food AB (assessed via a visual-probe task), energy intake (during a validated laboratory paradigm designed to simulate LOC-eating [46]) and brain activity (measured by magnetoencephalography) among adolescent girls with overweight or obesity. We hypothesized that following the smartphone program, girls who completed the AR program (vs a control program) would demonstrate a greater reduction in food AB scores, total energy intake, and intake of carbohydrates and fats, and increased intake of protein. We also hypothesized that following completion of the smartphone program, girls who completed the AR program would show decreased reward valuation of food cues via (1) decreased reward responsivity to food cues via increased oscillatory power in the striatum, ventral ACC and OFC during unconscious attention capture, and (2) increased attention control via decreased oscillatory power in the dorsal ACC, vlPFC, and dlPFC during attention deployment. We expected no change in the neural activity in any region of interest (ROI) among girls who completed the control program. Lastly, we explored recent LOC-eating as a moderator of the smartphone program’s effects on food AB, energy intake, and brain activity.

## 2. Materials and Methods

### 2.1. Study Design

Study procedures for this double-blind randomized control pilot trial for adolescent girls with overweight or obesity (Clinical Trials Identifier: NCT02977403) were approved by the National Institutes of Health (NIH) Institutional Review Board. All study visits occurred at the NIH Clinical Center. Participants were recruited by mail to local area parents, flyers posted at local public facilities, and Facebook advertisements. Interested participants were screened for eligibility over the phone and during an initial in-person visit. During the screening visit, informed consent and assent were obtained from parents and/or guardians and the participant, respectively. Participants completed a dual-energy X-ray absorptiometry (DXA) scan for body composition, a physical exam by a medical provider, self-report questionnaires, and an interview to determine the presence of recent LOC-eating. If deemed eligible at the screening visit, girls were randomized to complete either the AR or control program and were scheduled for a pre-intervention visit.

Participants were instructed to fast starting at 10 PM the night prior to the study visit. Adolescents arrived at the pre-intervention visit in the morning and completed a fasting blood draw, consumed a breakfast shake (17% protein, 16% fat, 67% carbohydrate) calibrated to their age, height, weight, and activity level, and underwent an MEG scan. Immediately following the scan, girls participated in a laboratory test meal. Lastly, participants were trained on how to use a provided smartphone. Starting the day following their pre-intervention visit, participants completed the (AR or control) smartphone program for two-weeks in their natural environment. After two weeks, girls returned to the NIH Clinical Center to complete a post-intervention assessment. The procedures and temporal order of study activities (i.e., breakfast shake, MEG scan, and test meal) during the post-intervention visit were the same as the pre-intervention visit. In addition, participants completed a single structural MRI scan (3T) during either their screening, pre-intervention, or post-intervention visit.

### 2.2. Participants

English speaking, right-handed, adolescent females with overweight or obesity (BMI, kg/m^2^ ≥ 85th percentile) aged 12–17 years were eligible to participate [47]. Exclusionary criteria were the presence of major medical illnesses (i.e., Cushing syndrome, untreated hypothyroidism) or a health condition that required medical treatment (i.e., hypertension or fasting hyperglycemia consistent with diabetes); use of medications known to affect body weight or eating behavior; current or past pregnancy; presence of any significant and full-threshold psychiatric disorder, except for binge-eating disorder; current and regular substance use; history of significant or recent brain injury; current involvement in treatment for weight loss or eating behavior; and presence of conditions where MEG is contraindicated (e.g., braces, metal implants).

### 2.3. Smartphone Program

Food-cue visual probe task: The AR and control programs were designed by one of the authors (AJW) and delivered using a smartphone application (“Colors”). The Colors smartphone application can administer several reaction time tasks and has been used successfully to administer an AR program for smoking reduction [48]. The food-cue visual probe task used in this study requires participants to complete several trials during which two colored photographs are presented side-by-side on a screen for 200 ms. Then, both images disappear and a probe (left or right pointing arrow) appears in a location previously occupied by one of the pictures. Participants are instructed to press a left button if the probe is pointing to the left, and a right button if the probe is pointing to the right, regardless of the side of the screen the probe appears on.

The smartphone program used high-palatability food (HF) stimuli and non-food (NF) stimuli, so that during all trials, participants viewed high-palatability food and non-food stimuli pairs (HF-NF). The only difference between AR and control programs was in the placement of the probe. For the experimental condition, the visual probe always replaced the non-food (neutral) stimulus. Thus, there was a perfect correlation between stimulus type and probe location. Individuals assigned to the AR program should, therefore, learn to attend away from the high-palatability food stimuli and identify the probe direction more quickly. For the control condition, the probe was equally likely to replace the food stimulus and the non-food stimulus. Therefore, there was no correlation between stimulus type and probe location, and no training of attention towards either food or non-food cues should occur. Consistent with previous attention retraining studies [48,49], the same program for AR and control conditions was used so that the duration of AR and control trials did not differ. This approach also ensured participants randomized to AR and control programs were exposed to the same food and neutral stimuli and received equal practice on the motoric aspects of the visual probe task.

Training frequency and timing: Daily, for two weeks, participants were prompted to complete three smartphone trainings, each consisting of 80 trials of the visual probe task. In addition to the trainings, participants completed a once-daily food AB assessment. The assessment required completion of 40 trials of the food-cue probe task. However, daily AB assessments were not part of the intervention program; thus, the probe replaced food and non-food images equally for all participants, regardless of treatment condition. Additionally, participants reported on their eating patterns and mood during each of the prompted trainings and AB assessments.

On school days, girls were prompted to complete AR (or control) trainings before school and immediately after school. They were also randomly prompted two more times between 3:30 pm and their bedtime, once for training and once to complete the daily AB assessment, which were completed in random order. Bedtime was set individually for each person at a time between 7:30 pm and 10:30 pm, based on the participant’s preference. On weekend days, the same schedule was used as on school days; however, participants were able to delay the first training by up to two hours to accommodate later waking times.

### 2.4. Outcome Measures

#### 2.4.1. Laboratory Test Meal

All participants received a multi-item, buffet-style meal (~11,000 kcal, 12% protein, 33% fat, 55% carbohydrate) comprising foods typically consumed by youth (e.g., chicken nuggets, white bread, turkey and ham, cheese slices, orange slices, carrots, tortilla chips, sandwich cookies, jellybeans). Prior to beginning the meal, girls were played tape-recorded instructions to “let yourself go and eat as much as you want”, and were left alone to eat. This is a well-validated paradigm which has been used in both adolescents [46] and adult [50] samples. The amount consumed at each meal was calculated by weighing each item before and after the test meal. Energy content and macronutrient composition consumed by participants was determined for each item according to data from the U.S.D.A. Nutrient Database for Standard Reference [51] or Food and Nutrient Database for Dietary Studies.

#### 2.4.2. Magnetoencephalography Scan

Brain magnetic fields were measured by a CTF 275 MEG system (CTF Systems, Inc., Coquitlam, BC, Canada) composed of a whole-head array of 275 SQUID sensors, in a magnetically shielded room (Vacuumschmelze, Hanau, Germany). Participants were in a seated position with the helmet placed around their heads. Head position within the magnetometer was determined by digitizing the position of the three indicator coils attached to the right and left preauricular and nasion fiducial points. Consistent with previous MEG research in pediatric samples [52,53], MEG data were sampled at 600 Hz (bandwidth 0–150 Hz).

Rest scan: At the beginning of the MEG session, participants completed a five-minute rest scan. Girls were instructed to keep their eyes open and focus on a black cross that was presented in the center of a white screen.

Food-cue visual probe task: Following the rest scan, participants completed 180 trials of the food-cue AB task. During the MEG scan, the images presented three types of stimuli: high-palatability food (HF), low-palatability food (LF), and non-food object (control stimuli, NF), with two differing stimuli simultaneously presented during each trial (on the right and on the left sides of the screen). Thus, there were three cue pairs (Figure S1; see Supplemental Materials): (1) HF-NF; (2) HF-LF; and (3) LF-NF. Each stimulus pair was presented 60 times, with the location of stimuli and location of the probe fully crossed. To minimize automaticity, the inter-trial interval was randomly jittered across three durations of 100 ms, 150 ms, and 500 ms. Additionally, to ensure participants understood the instructions, they engaged in a short practice session before completing the task. The practice trials resembled the main task, but no food stimuli were shown.

### 2.5. Other Measures

Anthropometric measures: Fasting weight was measured using a calibrated scale to the nearest 0.1 kg and height was measured in triplicate on a calibrated stadiometer to 0.1 cm. Weight and the average of three heights were used to calculate BMI, that was converted to BMI scores standardized for sex and age (BMIz) [54]. Total body fat mass (kg) was determined by a dual energy X-ray absorptiometry scan (GE Lunar iDXA, GE Healthcare, Madison WI; software GE encore 15), which is a validated body composition measure in youth [55].

Recent loss-of-control eating: The Eating Disorder Examination interview [56] is a semi-structured clinical interview of eating disorder psychopathology. Recent LOC-eating was determined as reporting at least one episode of LOC-eating (regardless of the amount of food consumed) in the prior 28 days. This interview has been found to be reliable and valid in adolescent samples [57,58,59]. All interviews were audio recorded. Twenty percent of the interviews conducted during the screening visit (*n* = 16) were reviewed and rated by a second, independent interviewer. There was 100% inter-rater agreement for presence of LOC-eating episodes in the prior month.

### 2.6. Sample Size Estimation

Sample size estimation was based on the power analysis for the first hypothesis (changes in AB scores following completion of the smartphone program). Meta-analytic studies of AR programs in adults found that AR programs produce large effects (*d* = 0.80–1.41) on reduction in AB and medium effects (*d* = 0.51–0.61) on target behaviors, such as smoking and eating [41,42]. Based on recommended equations for estimating sample size [60] when using general linear models, and assuming 35% attrition due to incomplete visual probe or MEG data [53,61,62], 80 girls, with a planned recruitment of 40 with LOC-eating and 40 without LOC-eating, were estimated to provide >80% power to detect medium to large effects.

### 2.7. Randomization and Blinding

In the current double-blind randomized control trial, girls were randomized to complete the AR or control smartphone program in blocks of eight with stratification for recent LOC-eating presence (LOC-eating or no LOC-eating), age (12–14 year or >14 year), and race (White or Other Race). Participants were each assigned a unique color and number combination to maintain blinding throughout the study. A study member who was not involved in data collection or analysis completed randomization and blinding procedures. Smartphone condition was not disclosed to participants at any point in the study. The unique codes (color, number) assigned to each participant were used to set up the smartphones, and maintain blinding of research coordinators involved in smartphone setup and data collection. Program allocation was revealed for data analysis only after all participants completed their pre- and post-intervention visits.

### 2.8. Analytic Plan

#### 2.8.1. Data Pre-Processing

AB reaction time scores: AB reaction time scores were derived from the dot probe task completed during the MEG scans. Reaction times scores were obtained for each of the stimulus pairings (3 total; HF-NF, LF-NF, HF-LF). Trials where the probe appeared behind the more salient food cue (e.g., a high-palatability food image, or low-palatability food image when the other image was a non-food image) were considered congruent trials. Trials where the probe appeared behind the less salient cue (e.g., non-food image, or low-palatability food image when the other image was a high-palatability food image) were considered incongruent trials. The participant’s average reaction time during incongruent trials was subtracted from the participant’s average reaction time during congruent trials. Thus, positive scores represent a quicker reaction time for (and bias towards) the more palatable stimulus, and negative scores represent a slower reaction time for (and bias away from) the more palatable stimulus. A difference score of 0 represents no bias towards or away from the more palatable stimulus. Consistent with prior studies, only trials where the participant responded correctly to the direction of the probe were included in computations.

Neural Oscillatory Power: Data processing was completed within MNE Python (v5.5.1) [63]. Data were structured into BIDS format using MNE-BIDS [64,65]. Standard MEG and MRI pre-processing steps were performed using Freesurfer (v7.4.1) [66] and the MNE-BIDS-Pipeline (v1.9) [67], including the following: cortical extraction and tessellation, source space generation (5 mm volumetric and 4096 nodes per hemisphere surface space), boundary element modeling, forward modeling, notch filtering (60/120/180 Hz), and epoching of the data. A structural MRI scan was co-registered to the MEG coordinate system. Three fiducial points (right and left preauricular and nasion) were marked via vitamin E capsules during the MRI scan to facilitate co-registration with MEG data. AFNI software (v24.04) [68] was used for co-registration with MEG data.

Additional processing was performed to generate the beamformer source localization using code developed for the study. Environmental noise was removed by applying reference channel third-order gradient compensation. Data were filtered from 13 to 35 Hz with a bandpass zero-phase FIR filter to extract the canonical beta band frequencies. The pre-stimulus baseline of 100 ms was used to generate the noise covariance matrix. The covariance matrix was generated using all data (0–500 ms following stimulus appearance). Linearly Constrained Minimum Variance (LCMV) beamformer weights were created using the data covariance. Trials were then segmented by (1) stimuli pairing (e.g., high-palatability food vs. non-food); (2) attention phase (capture = 0–250 ms following stimulus appearance, deployment = 250–500 ms following stimulus appearance); and (3) probe placement (i.e., congruent or incongruent trial). The segmented data were then used to create stimuli by timing by probe placement-specific covariance matrices and projected through the common covariance beamformer weights to generate surface and volumetric source reconstructions. Next, within each stimuli-pairing and attention phase, oscillatory power (pseudo-Z) during the incongruent trials was divided by oscillatory power during the congruent trials. The resulting difference value was then log transformed. The oscillatory power log scores were then averaged across all voxels within each a priori specified ROI. Volumetric model Freesurfer labels corresponding to regions in the striatum were obtained from the aseg atlas [69]. Surface model Freesurfer labels corresponding to regions in the ACC, OFC, dlPFC, and vlPFC were obtained from the aparc atlas [70]. This resulted in six oscillatory power measures (3 pairing types, 2 attention phases) in each ROI, for each participant at both pre-intervention and post-intervention.

#### 2.8.2. Hypothesis Testing

All analyses were conducted in python and are accessible on GitHub at https://github.com/Yanovski-Lab/AttentionRetraining_2weekOutcomes.git; (published on 19 July 2024). To examine changes in AB and oscillatory power from pre- to post-intervention, change scores were computed (∆ = post-intervention − pre-intervention) for AB reaction time scores and oscillatory power measures. Positive ∆ represents an increase in AB or oscillatory power from pre- to post-intervention. Negative ∆ represents a decrease in AB or oscillatory power from pre- to post-intervention. In general, there is an inverse association between the BOLD response and oscillatory power in the beta band; therefore, positive ∆_oscillatory power_ corresponds with decreased BOLD, and vice versa [71].

To compare the two conditions regarding change in food AB (∆_AB scores_) and neural activity (∆_oscillatory power_), linear mixed models were run with change scores as the dependent variable. Between-subject factors were condition, recent LOC-eating, and a term for the LOC-eating by condition interaction. AB and neural activity change scores were nested within subject. Models included a random intercept. Independent linear mixed models were run for ∆_oscillatory power_ in each a priori specified ROI, and for each attention phase (attention capture and attention deployment). All mixed models were adjusted for stimuli pairing (HF-NF, LF-NF, HF-LF), age, fat mass (kg) and height (cm) at pre-intervention, and race and ethnicity.

To test the effects of smartphone condition on energy intake, general linear models were used. Dependent variables were total caloric intake, and percentage intake from carbohydrates, fat, and protein. Models included condition as an independent variable, as well as a term for condition by LOC-eating interaction. Models were adjusted for age, fat mass (%), lean mass (kg), height (cm), race/ethnicity, and LOC-eating, as well as the respective intake variable (i.e., total calories, carbs, fat, protein) at the pre-intervention visit.

Participants who did not provide complete covariate data were excluded from analyses. The number of participants included in each outcome analysis varied, as participants were included in analyses only if they provided complete data on the outcome measure at both baseline and follow-up visits. Given the preliminary nature of the study, effect sizes and 95% confidence intervals, rather than statistical significance, were used to interpret changes in outcome measures. Cohen’s *d* values were computed to compare outcomes between the AR and control groups and were interpreted as minimal to no effect (<0.02), small effect (0.2–0.49), medium effect (0.5–0.79), and large effect (≥0.8) [60]. Cohen’s *d* values for the effect of condition were computed as:

$$\mathrm{Cohen}’s\;d=\frac{{EMM}_{active}-{EMM}_{control}}{{SD}_{pooled}}.$$

## 3. Results

### 3.1. Recruitment and Retention

**Figure 1:**
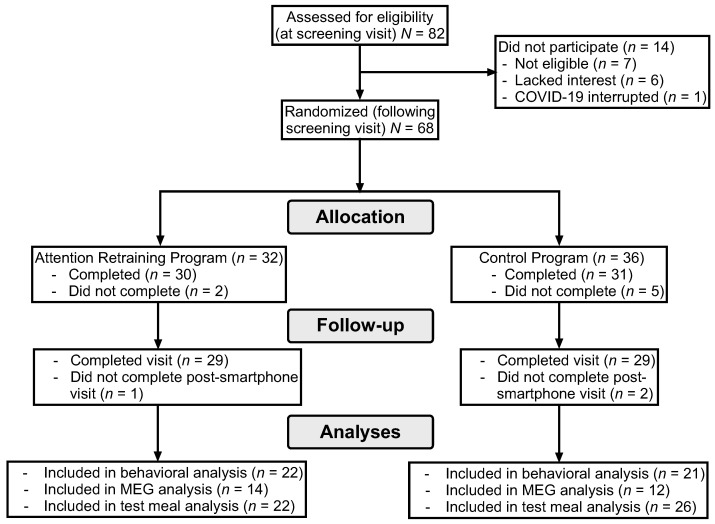
Consort diagram of participant retention.

Participants completed the study visits from February 2017 to September 2023. As shown in the consort diagram (Figure 1), 82 girls completed the screening visit. Of the 68 girls randomized to a smartphone program (*n*_AR_ = 32, *n*_control_ = 36), 7 did not complete it and an additional 3 girls did not complete the post-intervention visit. The demographic characteristics of girls randomized (*n* = 68) to the control and active conditions are reported in Table 1. Comparisons of participants who were and were not included in analyses are reported in the Supplemental Material. The NIH paused data collection and participant recruitment in March 2020, due to COVID-19 safety protocols. Study recruitment was concluded before the 80 were randomized due to a low recruitment rate following the onset of the COVID-19 pandemic.

### 3.2. ∆AB Scores Outcomes

**Figure 2:**
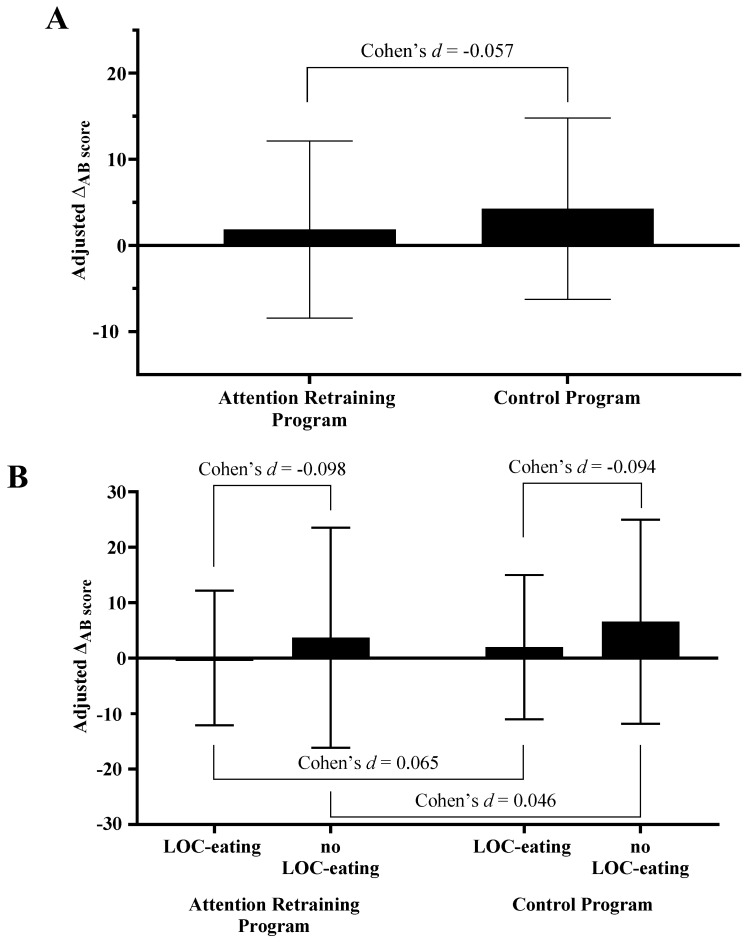
Effects of (**A**) condition and (**B**) condition by LOC-eating on change in ∆_AB score_. Note: LOC-eating; loss-of-control eating. Estimated marginal means (EMM) and 95% confidence intervals presented for the total sample (EMM_AR_ 1.85 [−8.44, 12.13]; EMM_control_ 4.27 [−6.25, 14.80]), AR group (EMM _LOC-eating_ 0.02 [−12.13, 12.18]; EMM _no LOC-eating_ 3.67 [−16.18, 23.52]), and Control group (EMM _LOC-eating_ 1.97 [−11.02, 14.97], EMM _no LOC-eating_ 6.57 [−11.81, 24.95]). The presented estimated marginal means for the effect of condition are from the models adjusted for LOC-eating and the LOC-eating × condition interaction term.

There was minimal to no effect of condition on changes in the visual probe AB task scores derived from the food-cue visual probe reaction times (β [95%CI] = −1.948 [−20.790, 16.894]; Cohen’s *d* = −0.057; Figure 2A). There was minimal to no interactive effect of LOC-eating by condition on ∆_AB score_ (β [95%CI] = −0.952 [−35.280, 33.377]; Cohen’s *d* = −0.098–0.165; Figure 2B).

### 3.3. Energy Intake Outcomes

The effects of condition by LOC-eating on energy intake are reported in Table 2 and Table 3, respectively. Unadjusted energy intake prior to and following completion of the smartphone program are reported in Supplemental Table S1. There was a small effect of condition on total energy intake, such that following the intervention the AR group consumed slightly more energy than the control group (*d* = 0.291). There was a medium effect of condition on carbohydrates (*d* = −0.544), with the AR group consuming a smaller percentage of energy from carbohydrates than the control group. There was also a medium effect of condition on fat (*d* = 0.615) and small effect of condition on protein (*d* = 0.216) intake with the AR group consuming a greater percentage of energy from these macronutrients than the control group (see Table 2).

Exploratory smartphone program × LOC-eating interaction effects: Within the control group, there was minimal to no effect of LOC-eating on energy intake (*d* = 0.101). However, there was a small effect of LOC-eating on carbohydrate intake (*d* = −0.288), minimal to no effect on fat intake (*d* = −0.023), and a medium effect on protein intake (*d* = 0.705), such that girls with LOC-eating consumed a somewhat greater percentage from carbohydrates and lower percentage from protein compared to girls without LOC-eating.

Within the AR group, there was minimal to no effect of LOC-eating on total energy intake (*d* = 0.067), carbohydrate intake (*d* = 0.046), fat intake (*d* = −0.005), nor protein intake (*d* = −0.095) following the intervention.

### 3.4. ∆_oscillatory power_ during Unconscious Attention Capture (0–250 ms)

**Figure 3:**
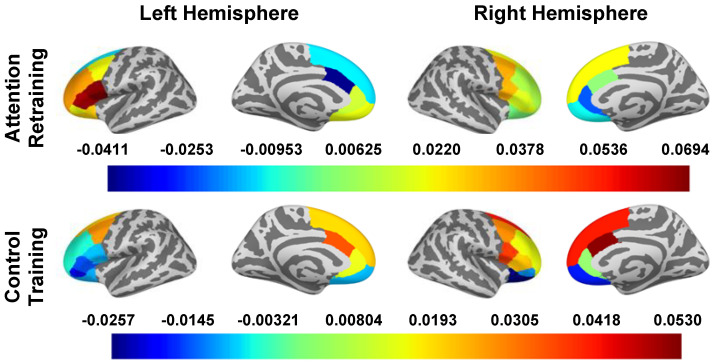
∆_oscillatory power_ in Surface ROIs During Attention Capture (0–250 ms). Note. Estimated marginal means of change (post intervention − pre intervention) in beta band oscillatory power are presented for all a priori identified ROIs. To obtain oscillatory power estimates, we log transformed ratios (pseudo-Z oscillatory power in congruent trials/pseudo-Z oscillatory power in incongruent trials); therefore, estimated marginal means are unitless. The presented estimated marginal means for the effect of condition are from the models adjusted for LOC-eating and the LOC-eating × condition interaction term.

The effect of the smartphone program on change in oscillatory power during attention capture are reported in Table 4 and Figure 3. In “bottom-up” regions, there were small to medium effects of condition on the left pallidum (*d* = 0.307), left putamen (*d* = 0.447), caudal ACC (*d*_left hemisphere_ = −0.569, *d*_right hemisphere_ = −0.397), right rostral ACC (*d* = −0.235), lateral OFC (*d*_left hemisphere_ = 0.291, *d*_right hemisphere_ = 0.207), and left medial OFC (*d* = 0.237). In general, the AR group had an increase (or smaller decrease) in oscillatory power in regions of the striatum and OFC while the control group had a decrease in oscillatory power among these regions. Additionally, the AR group had a decrease (or smaller increase) in oscillatory power in the rostral ACC while the control group had an increase in oscillatory power in this region.

Outcome of within-group changes in neural activity and the interactive effects of LOC-eating x condition on changes in neural activity during attention capture are reported in the Supplemental Material and in Supplemental Table S1.

### 3.5. ∆_oscillatory power_ during Attention Deployment (250–500 ms)

**Figure 4:**
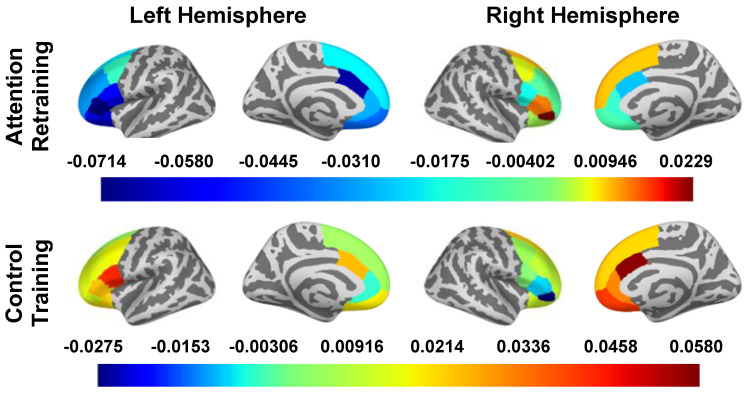
∆_oscillatory power_ in Surface ROIs During Attention Deployment (250–500). Note: Estimated marginal means of change (post intervention − pre intervention) in beta band oscillatory power are presented for all a priori identified ROIs. To obtain oscillatory power estimates, we log transformed ratios (pseudo-Z oscillatory power in congruent trials/pseudo-Z oscillatory power in incongruent trials); therefore, estimated marginal means are unitless. The presented estimated marginal means for the effect of condition are from the models adjusted for LOC-eating and the LOC-eating × condition interaction term.

The main effects of the smartphone program on the change in oscillatory power during attention deployment are reported in Table 5 and Figure 4. In “top-down” regions, there were small to large effects of condition on the caudal ACC (d left hemisphere = −0.817, *d* right hemisphere = −0.594), left dlPFC (*d* caudal = −0.228, *d* rostral = −0.472, *d* superior = −0.286), and left vlPFC (*d* pars opercularis = −0.802, *d* pars orbitalis = −0.545, *d* pars triangularis = −0.805). The AR group had a decrease in oscillatory power among these regions, except for the right pars orbitalis, where increased oscillatory power was observed. The control group had an increase in oscillatory power in these regions, except for in the left pallidum and right pars orbitalis, where decreased oscillatory power was observed.

The outcome of within-group changes in neural activity and the interactive effects of LOC-eating × condition on changes in neural activity during attention deployment are reported in the Supplemental Material and in Supplemental Table S2.

### 3.6. Adverse Events

All participants completed the smartphone program (AR or control) they were randomized to complete. No adverse events were reported during completion of the smartphone program. However, adverse events occurred during the MEG scans: nausea or vomiting (three participants in the AR group), headache (one participant in the control group and one participant in the AR group), and sore neck (one participant in the control group).

## 4. Discussion

This pilot, double-blind, randomized control trial investigated whether a two-week long, smartphone-delivered AR program (versus a control program) altered food AB in adolescent girls with overweight or obesity. Our findings provide preliminary support for the potential effectiveness of an intensive, smartphone-delivered food AR program to alter neural activity associated with attention processing and changes in eating behavior. Specifically, our AR program did not promote significant changes in AB as measured by a food-cue visual probe task, but produced detectable changes in neural correlates of attention capture and deployment. The observed changes in neural activity broadly indicate a slightly blunted initial reward response, reduced stimulus-driven attention, and increased goal-directed attention and action control. The AR program also promoted less carbohydrate intake in the laboratory. However, greater fat and protein intake as a percentage of energy offset this reduction and resulted in slightly greater total energy intake. However, no changes in energy intake were clinically significant.

We observed no statistically significant change in (reaction time-based) food AB scores among girls who completed the AR or control programs. These results are contrary to our hypothesis and reported outcomes from prior food-based AR interventions [37]. Although reaction time scores are the most common outcome measure of AR programs [37,72], they have several limitations, including reliance on discrete events (when a stimulus is shown) rather than continuous assessment of shifts in attention [73]; potential influence by non-attention specific processes, such as response execution [74]; and poor psychometric reliability and stability [75,76], which likely introduced noise into our reaction time assessments. To improve outcome measurement of food AR programs, future studies could employ multiple approaches to assess attention processes, such as using a behavioral task and eye tracking, as well as computational modeling [74,77].

Following completion of the smartphone program there was a modest difference (~71 kcal) in energy intake and fat intake (2.4%) between the AR and control groups. The difference in energy intake was opposite to what we hypothesized. Our total energy intake results are consistent with results from other food AB programs, which have failed to consistently produce improvements in appetite and eating behavior [37]. However, consistent with hypotheses, we observed a lesser consumption of percentage intake from carbohydrates and a greater percentage of energy intake from protein among the AR group compared to the control group. Exploratory analyses showed that girls in the control condition with LOC-eating consumed more carbohydrates and lesser protein than their counterparts without LOC-eating, which is consistent with the phenotypic eating behavior of adolescents with LOC-eating [46,78]. However, energy and macronutrient intake did not differ between girls with and without LOC-eating who completed the AR program. The observed pattern of macronutrient intake among the AR group, regardless of LOC-eating status, suggest our AR program might produce increased control of energy intake by promoting foods higher in protein and lower in carbohydrates, rather than reducing self-served portion sizes. Studies with longer follow-up periods and are needed to determine whether these changes are generalizable to eating episodes that occur in naturalistic environments.

We observed changes in brain regions among girls that completed the AR program that are suggestive of reduced biases in attentional processing. Among the AR group during attention capture, there were no changes in activity in the caudate, pallidum, ventral ACC or OFC, but there was decreased engagement of the left putamen. The putamen is a region of the striatum associated with stimulus-reward associations [79]. Consistent with our hypotheses, we observed more robust changes among the AR group during attention deployment. Specifically, among the AR group (compared to the control group) we observed (1) relatively lower engagement in regions of the vlPFC and OFC and (2) increased engagement of the pallidum and OFC. These changes are suggestive of greater direction of attention towards goal-related stimuli [80,81,82], stimulus–outcome action learning [83,84], and goal-directed decision making [85,86]. These findings are particularly promising because the opposite pattern of activation in these areas has been associated with having a higher BMI and/or future increases in BMI [11]. Interestingly, although attention processes are thought to be lateralized to the right hemisphere of the brain [87,88], most of the changes among our AR group occurred in the left hemisphere of the brain. Greater engagement in left hemisphere might support attentional control by promoting greater goal-driven and object-based orienting of attention [89,90]. Therefore, the AR program appears to have largely bolstered the engagement of attention support systems.

The effects of the AR program were somewhat different among girls with and without LOC-eating. During attention capture, girls in the AR condition with LOC-eating experienced minimal to no change in oscillatory power in any ROI. Thus, the observed changes in neural activity among girls who completed the AR program appeared to be driven by girls without LOC-eating. However, there was no clear pattern for the effect of LOC-eating on neural outcomes during attention deployment for the AR group. Changes in brain regions associated with reward-related motivation [91,92], stimulus–outcome action learning [83,84], and stimulus-directed reorienting [85,93] seem to be driven by girls with LOC-eating. Alternatively, changes in goal-directed attention [86] might be driven by girls without LOC-eating. Changes in impulsivity and attentional switching [80,81] were observed in both girls with and without LOC-eating. The pattern of results might suggest that girls with LOC-eating have greater difficulty suppressing reward response and stimulus-driven attention compared to girls without LOC-eating. These differences in changes in neural activity did not correspond to differences in AB reaction time scores or energy intake. Thus, there may be different neural pathways for reducing the impact of food AB on energy intake among people with and without LOC-eating. Studies with larger samples of girls with LOC-eating are needed to bolster support for this interpretation.

Consistent with our hypotheses, the control smartphone program produced minimal to no changes in neural activity. Following completion of the smartphone program, the control group had no changes in neural activity associated with reward valuation or responsivity during attention capture. During attention deployment, the control group had decreased engagement in some brain regions associated with direction of attention towards goal-related stimuli [82], and an updating of stimulus–reward associations [83,84] and goal-driven attention and action selection [23,94,95]. Additionally, exploratory interaction analyses revealed that girls in the control condition with LOC-eating had lower engagement in most ROIs during both attention capture and deployment. The observed decreases in inhibition and goal-oriented attention were likely driven by girls with recent LOC-eating during attention deployment. Thus, in the absence of any AR intervention, girls with LOC-eating are likely to continue to exhibit a greater vulnerability to food AB.

In general, findings from this study provide tentative support for Attentional Control models of AB modification [33]. Attentional Control Models assert that observed reductions in inhibitory control and increased goal-oriented action, regardless of reward responsivity, promote decreased AB [33], and subsequently would improve energy intake. Alternatively, Valence-Specific Models suggest blunting of the reward response drives reductions in AB and associated behaviors [33]. Response patterns among the AR group seem to provide more support for Action Control models. Specifically, during attention capture we observed reduced engagement only in the left putamen, and during attention deployment we saw increased engagement in regions of the striatum, ventral ACC, and OFC. Additionally, the response pattern observed among girls who completed the control program provides additional support for Attentional Control Models. The control group experienced a decreased engagement in some brain regions associated with reward responsivity and inhibitory control. Girls in the control group also consumed a greater percentage of energy from carbohydrates and less from protein during a laboratory meal than the AR group following completion of the smartphone program. This pattern is in contrast with Valence-Specific Models, but consistent with Attention Control models. Some recent research among adults also supports the notion that increasing attentional control (e.g., through cognitive reappraisal) reduces food AB [96]. However, additional research is needed to map theory and neural mechanisms of change onto behavioral outcome from food AR programs.

This study has several strengths. We recruited a sample of racially and ethnically diverse girls, potentially increasing the generalizability of findings to diverse populations. LOC-eating was assessed by a validated interview and energy consumption with an in-laboratory feeding paradigm. We used DXA to assess body composition, which is a more appropriate measure of adiposity than BMI [97]. We also used MEG, an ideal neuroimaging methodology for measuring the minute temporal changes that underly attention processing [62,98]. The high temporal acuity of MEG scans allowed us to gain a more in-depth understanding of food AB processes by disaggregating magnetic field changes during attention capture and deployment phases. A limitation of this study is its small sample size, such as that of the participants who responded positively regarding occurrences of LOC-eating. Additionally, participants who provided complete data for MEG analyses were, on average, older than participants who were not included in MEG analyses. A large percentage of MEG data were missing due to head motion, adverse events (e.g., feeling sick), and technical issues during data collection; common challenges experienced when collecting MEG scans of young people [99,100].

## 5. Conclusions

Findings from this pilot double-blind randomized clinical trial provide preliminary evidence that a 2-week smartphone AR program promoted changes in food choices leading to a decreased percentage of carbohydrate intake during a laboratory meal designed to induce LOC-eating. This change may be due to alterations in neural activity consistent with blunted reward responsivity during unconscious attention capture and increased attentional control and goal-directed action during attention deployment towards food cues. This preliminary study increases our understanding of the mechanisms involved in the association between food AB and aberrant eating behavior. However, additional research with larger samples is needed to demonstrate the validity of our findings and determine the optimal frequency and duration of food AR programs to maximize positive outcomes and minimize burdens.

## Figures and Tables

**Table 1:** Characteristics of participants randomized to control and attention retraining programs.

Characteristic	Total Sample (*N* = 68)	Control Program (*n* = 36)	AR Program (*n* = 32)	Comparisons
				*t*, *p*
Age (year) ^1^	14.93 ± 1.64	14.88 ± 1.70	15.00 ± 1.61	−0.31, 0.76
BMIz ^1^	1.84 ± 0.60	1.88 ± 0.64	1.80 ± 0.55	0.54, 0.59
Fat mass (kg) ^1^	33.71 ± 10.79	33.64 ± 11.31	33.79 ± 10.36	−0.06, 0.95
Height (cm) ^1^	161.89 ± 6.46	161.29 ± 6.58	162.58 ± 6.35	−0.82, 0.42
				χ^2^, *p*
Recent LOC-eating ^2^	20 (29.41)	11 (30.56)	9 (28.13)	0.05, 0.83
Race ^2^				3.18, 0.37
Asian	1 (1.5)	1 (2.8)	0 (0)	
Black	37 (54.4)	19 (52.8)	18 (56.3)	
Multiracial	7 (10.3)	2 (5.5)	5 (15.6)	
White	23 (33.8)	14 (38.9)	9 (28.1)	
Ethnicity ^2^				0.97, 0.62
Hispanic	9 (13.2)	6 (16.7)	3 (9.4)	
Non-Hispanic	54 (79.4)	27 (75.0)	27 (84.4)	
Unreported	5 (7.4)	3 (8.3)	2 (6.2)	

Note. BMIz: body mass index adjusted for age and sex; recent LOC-eating: presence of 1 or more reported loss-of-control eating episodes as identified by the Eating Disorder Interview. One subject randomized to the active condition had missing baseline height and therefore BMIz data. ^1^ Mean ± Standard Deviation. ^2^ *n* (%).

**Table 2:** Effect of condition on energy intake.

	Effect of Condition	Control (*n* = 26)	AR (*n* = 22)	Effect Size
	β [95%CI]	EMM [95%CI]	EMM [95%CI]	Cohen’s *d*
Total Calories (kcal)	66.322 [−133.345–265.988]	1017.024 [923.141–1110.906]	1088.188 [986.126–1190.249]	0.291 *
Carbohydrate (%)	−0.021 [−0.064–0.023]	78.3 [76.3–80.4]	75.4 [73.2–77.6]	−0.544 **
Fat (%)	0.024 [−0.008–0.057]	65.0 [63.5–66.5]	67.4 [65.8–69.1]	0.615 **
Protein (%)	−0.006 [−0.032–0.020]	37.3 [36.1–38.6]	38.0 [36.7–39.4]	0.216 *

Note. * small effect Cohen’s *d* = 0.20; ** medium effect Cohen’s *d* = 0.50. β: beta; EMM: estimated marginal mean; CI: confidence interval. β derived from general linear models adjusted for age, fat mass (%), lean mass (kg), height (cm), race and ethnicity (0 = non-Hispanic White, 1 = other race or ethnicity), and LOC-eating (0 = absent, 1 = present), as well as the respective intake variable (i.e., total energy intake, carbs, fat, protein) at baseline. The presented estimated marginal means for the effect of condition are from the models adjusted for LOC-eating and the LOC-eating × condition interaction term. Percentages were arcsin(sqrt)-transformed before analysis, so percentages do not add up to 100%. See Supplemental Materials for untransformed results.

**Table 3:** Interactive effect of condition and LOC-eating on energy intake.

	Effect of Condition × LOC	Control No LOC (*n* = 16)	Control LOC (*n* = 10)	Control No LOC–LOC	AR No LOC (*n* = 15)	AR LOC (*n* = 7)	AR No LOC–LOC
	β [95%CI]	EMM [95%CI]	EMM [95%CI]	*d*	EMM [95%CI]	EMM [95%CI]	Cohen’s *d*
Total Calories (kcal)	9.684 [−335.492–354.860]	1029.919 [907.552–1152.286]	1004.128 [849.345–1158.912]	0.101	1096.241 [969.861–1222.621]	1080.134 [895.132–1265.136]	0.067
Carbohydrate (%)	−0.017 [−0.092–0.058]	77.6 [74.9–80.3]	79.0 [75.6–82.4]	−0.288 *	75.5 [72.8–78.3]	75.3 [71.2–79.3]	0.046
Fat (%)	−0.001 [−0.056–0.055]	65.0 [66.9–63]	65.1 [67.5–62.6]	−0.023	67.4 [69.5–65.4]	67.4 [70.4–64.5]	−0.005
Protein (%)	0.026 [−0.019–0.071]	38.5 [36.9–40.1]	36.2 [34.1–38.2]	0.705 **	37.9 [36.2–39.5]	38.2 [35.7–40.6]	−0.095

Note. * small effect Cohen’s *d* = 0.20; ** medium effect Cohen’s *d* = 0.50. β: beta; EMM: estimated marginal mean; CI: confidence interval. β derived from general linear models adjusted for age, fat mass (%), lean mass (kg), height (cm), race and ethnicity (0 = non-Hispanic White, 1 = other race or ethnicity), and LOC-eating (0 = absent, 1 = present), as well as the respective intake variable (i.e., total energy intake, carbs, fat, protein) at baseline. The presented estimated marginal means for the effect of condition are from the models adjusted for LOC-eating and the LOC-eating × condition interaction term. Percentages were arcsin(sqrt)-transformed before analysis, so percentages do not add up to 100%. See Supplemental Materials for untransformed results.

**Table 4:** Effects of treatment condition on ∆oscillatory power during attention capture (0–250 ms following stimulus).

	Effect of Condition	Control (*n* = 12)	AR (*n* = 14)	Effect Size
ROI Sub-Regions	β [95%CI]	EMM [95%CI] ^a,b^	EMM [95%CI] ^a,b^	Cohen’s *d*
Striatum				
Caudate-lh	0.047 [−0.025–0.119]	0.010 [−0.027–0.048]	0.021 [−0.014–0.056]	0.093
Caudate-rh	−0.002 [−0.085–0.082]	0.032 [−0.011–0.075]	0.023 [−0.017–0.063]	−0.066
Pallidum-lh	0.08 [−0.022–0.182]	−0.007 [−0.055–0.041]	0.038 [−0.006–0.083]	0.307 *
Pallidum-rh	0.013 [−0.086–0.113]	0.028 [−0.022–0.078]	0.009 [−0.037–0.056]	−0.121
Putamen-lh	0.083 [−0.001–0.167]	−0.013 [−0.056–0.03]	**0.046 [0.006–0.086]**	0.447 *
Putamen-rh	0.031 [−0.059–0.121]	0.017 [−0.029–0.063]	0.02 [−0.022–0.063]	0.020
ACC				
Caudal-lh	−0.042 [−0.149–0.065]	0.035 [−0.009–0.078]	**−0.041 [−0.081–−0.001]**	−0.569 **
Caudal-rh	−0.034 [−0.122–0.053]	**0.053 [0.011–0.095]**	0.002 [−0.037–0.041]	−0.397 *
Rostral-lh	0.042 [−0.041–0.126]	0.012 [−0.03–0.055]	0.012 [−0.027–0.051]	−0.003
Rostral-rh	0.021 [−0.06–0.102]	0.002 [−0.034–0.039]	−0.024 [−0.058–0.01]	−0.235 *
OFC				
Lateral-lh	**0.107 [0.03–0.185]**	−0.010 [−0.050–0.030]	0.026 [−0.011–0.063]	0.291 *
Lateral-rh	**0.092 [0.01–0.175]**	−0.026 [−0.065–0.014]	−0.001 [−0.037–0.036]	0.207 *
Medial-lh	0.046 [−0.032–0.123]	−0.010 [−0.048–0.029]	0.018 [−0.017–0.054]	0.237 *
Medial-rh	0.041 [−0.03–0.112]	−0.018 [−0.053–0.018]	−0.011 [−0.044–0.022]	0.062
dlPFC				
Caudal-lh	−0.037 [−0.125–0.052]	0.026 [−0.006–0.058]	0.02 [−0.01–0.049]	−0.068
Caudal-rh	0.001 [−0.091–0.093]	0.027 [−0.010–0.065]	0.031 [−0.003–0.066]	0.037
Rostral-lh	0.065 [−0.001–0.132]	−0.006 [−0.040–0.029]	**0.033 [0.001–0.066]**	0.365 *
Rostral-rh	0.023 [−0.055–0.101]	0.014 [−0.018–0.046]	0.008 [−0.021–0.038]	−0.061
Superior-lh	−0.032 [−0.116–0.051]	0.021 [−0.004–0.046]	−0.013 [−0.036–0.01]	−0.438 *
Superior-rh	−0.012 [−0.078–0.053]	**0.041 [0.017–0.064]**	0.019 [−0.002–0.041]	−0.294 *
vlPFC				
Pars opercularis-lh	**0.089 [0.004–0.174]**	−0.010 [−0.048–0.029]	**0.069 [0.034–0.105]**	0.672 **
Pars opercularis-rh	−0.016 [−0.113–0.081]	0.037 [−0.007–0.08]	0.031 [−0.009–0.071]	−0.045
Pars orbitalis-lh	**0.129 [0.049–0.209]**	−0.019 [−0.060–0.023]	0.019 [−0.019–0.058]	0.299 *
Pars orbitalis-rh	**0.126 [0.045–0.207]**	−0.011 [−0.053–0.030]	0.016 [−0.023–0.055]	0.214 *
Pars triangularis-lh	**0.115 [0.027–0.203]**	−0.015 [−0.059–0.029]	**0.064 [0.024–0.105]**	0.589 **
Pars triangularis-rh	0.037 [−0.053–0.127]	0.022 [−0.020–0.063]	0.016 [−0.023–0.054]	−0.047

Note. * small effect Cohen’s *d* = 0.20; ** medium effect Cohen’s *d* = 0.50.. ROI: region of interest; β: beta; EMM: estimated marginal mean; AR: attention retraining; -lh: left hemisphere; -rh: right hemisphere; ACC: anterior cingulate cortex; OFC: orbitofrontal cortex; dlPFC: dorsolateral prefrontal cortex; vlPFC: ventrolateral prefrontal cortex. Estimated marginal mean represents the group-level mean change score (post intervention − pre intervention) of beta band power. ^a^ A decrease in power corresponds to an increase in activity. Thus, a negative estimated marginal mean reflects increased activity in that brain region post-intervention. Thus, a negative estimated marginal mean reflects increased activity in that brain region post-intervention. ^b^ A positive estimated marginal mean reflects decreased activity in that brain region post-intervention. Estimated marginal means with a 95%CI that does not contain 0 are in bolded font. Linear mixed models were adjusted for stimuli pairing (HF-NF, LF-NF, HF-LF), age, fat mass (kg) and height (cm) at pre-intervention, race and ethnicity (0 = non-Hispanic White, 1 = other race or ethnicity), and LOC-eating (0 = absent, 1 = present), and included a LOC-eating × condition interaction term.

**Table 5:** Effects of treatment condition on ∆oscillatory power during attention deployment (250–500 ms following stimulus).

	Effect of Condition	Control (*n* = 12)	AR (*n* = 14)	Effect Size
ROI Sub-Regions	β [95%CI]	EMM [95%CI] ^a,b^	EMM [95%CI] ^a,b^	Cohen’s *d*
Striatum				
Caudate-lh	−0.021 [−0.092–0.05]	0.012 [−0.025–0.049]	**−0.052 [−0.086–−0.017]**	−0.561 **
Caudate-rh	−0.013 [−0.095–0.069]	0.030 [−0.013–0.073]	−0.031 [−0.07- 0.009]	−0.464 *
Pallidum-lh	−0.005 [−0.103–0.092]	−0.011 [−0.058–0.036]	**−0.051 [−0.095–−0.008]**	−0.281 *
Pallidum-rh	0.02 [−0.069–0.109]	0.037 [−0.009–0.084]	−0.026 [−0.069–0.017]	−0.445 *
Putamen-lh	−0.01 [−0.099–0.08]	<0.001 [−0.040–0.041]	**−0.062 [−0.10–−0.025]**	−0.505 **
Putamen-rh	0.013 [−0.069–0.095]	0.018 [−0.024–0.061]	−0.031 [−0.07–0.009]	−0.375 *
ACC				
Caudal-lh	−0.041 [−0.123–0.041]	0.026 [−0.012–0.064]	**−0.069 [−0.104–−0.033]**	−0.817 ***
Caudal-rh	−0.038 [−0.142–0.066]	**0.058 [0.011–0.105]**	−0.028 [−0.072–0.016]	−0.594 **
Rostral-lh	−0.023 [−0.105–0.059]	−0.005 [−0.048–0.038]	−0.03 [−0.07–0.01]	−0.189
Rostral-rh	−0.057 [−0.14–0.027]	0.032 [−0.010–0.074]	−0.012 [−0.051–0.027]	−0.341 *
OFC				
Lateral-lh	0 [−0.091–0.091]	0.021 [−0.022–0.063]	**−0.062 [−0.102–−0.023]**	−0.641 **
Lateral-rh	−0.01 [−0.092–0.071]	0.012 [−0.029–0.052]	0.002 [−0.035–0.04]	−0.075
Medial-lh	−0.013 [−0.101–0.076]	0.018 [−0.023–0.058]	**−0.038 [−0.076–−0.001]**	−0.454 *
Medial-rh	−0.033 [−0.11–0.043]	**0.041 [0.001–0.080]**	−0.009 [−0.046–0.028]	−0.404 *
dlPFC				
Caudal-lh	0.011 [−0.069–0.091]	0.016 [−0.024–0.056]	−0.012 [−0.049–0.025]	−0.228 *
Caudal-rh	0.054 [−0.037–0.146]	0.005 [−0.041–0.050]	0.006 [−0.036–0.048]	0.008
Rostral-lh	0.002 [−0.061–0.065]	0.015 [−0.017–0.047]	**−0.031 [−0.061–−0.002]**	−0.472 *
Rostral-rh	0.032 [−0.05–0.115]	0.010 [−0.024–0.044]	−0.006 [−0.037–0.026]	−0.149
Superior-lh	−0.016 [−0.075–0.042]	0.004 [−0.025–0.033]	−0.022 [−0.049–0.005]	−0.286 *
Superior-rh	0.03 [−0.039–0.099]	0.023 [−0.009–0.055]	0.01 [−0.02–0.039]	−0.138
vlPFC				
Pars opercularis-lh	−0.016 [−0.097–0.066]	**0.043 [0.003–0.082]**	**−0.054 [−0.09–−0.017]**	−0.802 ***
Pars opercularis-rh	0.053 [−0.051–0.156]	0.001 [−0.037–0.039]	−0.018 [−0.053–0.017]	−0.163
Pars orbitalis-lh	0.015 [−0.061–0.091]	0.013 [−0.026–0.053]	**−0.052 [−0.089–−0.016]**	−0.545 **
Pars orbitalis-rh	0.036 [−0.047–0.119]	−0.027 [−0.067–0.012]	0.023 [−0.014–0.06]	0.412 *
Pars triangularis-lh	−0.017 [−0.092–0.057]	0.023 [−0.015–0.062]	**−0.071 [−0.107–−0.036]**	−0.805 ***
Pars triangularis-rh	0.07 [−0.019–0.159]	−0.009 [−0.055–0.037]	0.013 [−0.03–0.056]	0.156

Note. * small effect Cohen’s *d* = 0.20; ** medium effect Cohen’s *d* = 0.50; *** large effect Cohen’s *d* = 0.80. ROI: region of interest; β: beta; EMM: estimated marginal mean; AR: attention retraining; -lh: left hemisphere; -rh: right hemisphere; ACC: anterior cingulate cortex; OFC: orbitofrontal cortex; dlPFC: dorsolateral prefrontal cortex; vlPFC: ventrolateral prefrontal cortex. Estimated marginal mean represents the group-level mean change score (post intervention − pre intervention) of beta band power. ^a^ A decrease in power corresponds to an increase in activity. Thus, a negative estimated marginal mean reflects increased activity in that brain region post-intervention. ^b^ A positive estimated marginal mean reflects decreased activity in that brain region post-intervention. Estimated marginal means with a 95%CI that does not contain 0 are in bolded font. Linear mixed models were adjusted for stimuli pairing (HF-NF, LF-NF, HF-LF), age, fat mass (kg) and height (cm) at pre-intervention, race and ethnicity (0 = non-Hispanic White, 1 = other race or ethnicity), and LOC-eating (0 = absent, 1 = present), and included a LOC-eating × condition interaction term.

## Data Availability

Data are available at https://github.com/Yanovski-Lab/AttentionRetraining_2weekOutcomes.git (published on 19 July 2024).

## Supplementary Materials

The following supporting information can be downloaded at: https://www.mdpi.com/article/10.3390/nu16203456/s1, Figure S1: Schematic of the food-cue visual probe task; Table S1: Unadjusted energy intake prior to and following completion of the smartphone program; Table S2: Interactive effects of condition and LOC-eating on ∆_oscillatory power_ during attention capture (0–250 ms following stimulus); Table S3: Interactive effects of condition and LOC-eating on ∆_oscillatory power_ during attention deployment (250–500 ms following stimulus).
