# Supplementary material for: A Pilot Randomized Control Trial Testing a Smartphone-Delivered Food Attention Retraining Program in Adolescent Girls with Overweight or Obesity
Source: Nutrients. 2024 Oct 12;16(20):3456. doi: 10.3390/nu16203456 (PMC11510407; doi:10.3390/nu16203456)
Supplement: Supplementary file 1 [file nutrients-16-03456-s001.zip › nutrients-3233536-supplementary.pdf]

Article

A pilot randomized control trial testing a smartphone delivered food attention retraining program in adolescent girls with overweight or obesity

Megan N. Parker,[1,2†] Bess F. Bloomer,[1†] Jeffrey D. Stout,[3] Meghan E. Byrne,[4] Natasha A. Schvey,[1,2] Sheila M. Brady,[1] Kong Y. Chen,[5] Allison C. Nugent,[3] Sara A. Turner[6], Shanna B. Yang,[6] Monika M. Stojek,[7] Andrew J. Waters,[2] Marian Tanofsky-Kraff,[1,2††*]& Jack A. Yanovski[1††*]

1Section on Growth and Obesity, Division of Intramural Research, *Eunice Kennedy Shriver* National Institute of Child Health and Human Development (NICHD), National Institutes of Health (NIH), Department of Health and Human Services (DHHS), 10 Center Drive, Room 1-3330, Bethesda, MD, 20892-1103, USA
2Department of Medical and Clinical Psychology, Uniformed Services University of the Health Sciences (USUHS), 4301 Jones Bridge Road, Bethesda, MD, 20814, USA
3MEG Core Facility, National Institute of Mental Health (NIMH), Bethesda, MD, USA
4Section on Development and Affective Neuroscience, Emotion and Development Branch, National Institute of Mental Health (NIMH), Bethesda, MD, USA
5Diabetes, Endocrinology, and Obesity Branch, Division of Intramural Research, National Institute of Diabetes and Digestive and Kidney Diseases, National Institutes of Health, Bethesda, MD, USA
6Nutrition Department, Clinical Center, NIH, 10 Center Drive, Bethesda, MD, 20892, USA
7Institute of Psychology, University of Silesia in Katowice, Poland

†co-first authors, ††co-senior authors

*Correspondence to: Jack A. Yanovski, MD, PhD, Chief of the Section on Growth and Obesity, Division of Intramural Research, Eunice Kennedy Shriver National Institute of Child Health and Human Development, NIH, 10 Center Drive, Hatfield Clinical Research Center, Room 1-3330, Bethesda, MD, 20892-1103; Email: yanovskj@mail.nih.gov; Phone: (301)-496-0858 and Marian Tanofsky-Kraff, PhD, Professor, Department of Medical and Clinical Psychology and Medicine, USU, 4301 Jones Bridge Road, Bethesda, MD, 20814; Email: marian.tanofsky-kraff@usuhs.edu; Phone: 301-295-1482

Supplemental Results

Missing Data

Girls who completed the intervention and post-intervention visit (n = 58) did not differ from girls who did not complete the intervention or post-intervention visit (n=10) on age (t = -1.08, p = .29), BMIz (t = 0.78, p = .44), race and ethnicity ($\chi 2$ = 0.99, p = .32) or presence of LOC-eating ($\chi 2$ = 0.50, p = .48). Of note, only some of the 58 participants who completed post-intervention visits had sufficiently complete MEG (n = 26, 45%), visual probe task (n = 43, 74%) and test meal (n = 48, 83%) data to be included in analyses. Girls who did provide complete MEG data were older (t = -2.26, p = .01) and had a lower BMIz (t = 2.69, p = .01) than girls who did not provide complete MEG data. However, girls who did and did not provide complete MEG data did not differ on race and ethnicity ($\chi 2$ = 1.86, p = .17) or presence of LOC-eating ($\chi 2$ = 0.13, p = .72). Girls who did and did not provide complete visual probe AB task data did not differ on age (t = -1.85, p = .07), BMIz (t = 0.17, p = .86), race and ethnicity ($\chi 2$ = 0.84, p = .36), or presence of LOC-eating ($\chi 2$ = 0.38, p = .85). Girls who did and did not provide complete test meal data did not differ on age (t = -0.19, p = .85), BMIz (t = 0.28, p = .78), race and ethnicity ($\chi 2$ = 0.82, p = .37), or presence of LOC-eating ($\chi 2$ = 2.84, p = .09).

Energy intake outcomes

Unadjusted intake prior to and following completion of the smartphone program are reported in Supplemental Table 1.

$\Delta_{\text{oscillatory power}}$ during unconscious attention capture (0-250ms)

Control group. Among the control group, there was an increase in oscillatory power among the right caudal ACC (EMM[95%CI] = 0.053 [0.011- 0.095]) and right superior dlPFC (EMM[95%CI] = 0.041 [0.017 - 0.064]).

AR group. The AR group had a decrease in oscillatory power among the left caudal ACC (EMM[95%CI] = -0.041 [-0.081- -0.001]), and increased oscillatory power in the left putamen (EMM[95%CI] = 0.046 [0.006- 0.086]), left rostral dlPFC (EMM[95%CI] = 0.033 [0.001- 0.066]), left pars opercularis (0.069 [0.034 - 0.105]) and left pars triangularis (EMM[95%CI] = 0.064 [0.024- 0.105]).

Exploratory interaction effects of smartphone program and LOC-eating. The interactive effects of smartphone program and LOC-eating on change in oscillatory power during attention capture are reported in Supplemental Table 2.

Control group. Girls in the control group without LOC-eating had an increase in left caudal dlPFC (EMM[95%CI] = 0.04 [0 - 0.08]) and decrease in left pars orbitalis (EMM[95%CI] = -0.065 [-0.116 - -0.013]). While the girls in the control group with LOC-eating had an increase in the right superior dlFC (EMM[95%CI] = 0.060 [0.019 - 0.101]). Within the control group, there were small to medium effects of LOC-eating on oscillatory power in the pallidum ($d_{\text{left hemisphere}}$ =-0.374, $d_{\text{right hemisphere}}$ = -0.336) and putamen ($d_{\text{left hemisphere}}$ =-0.328, $d_{\text{right hemisphere}}$ = -0.261), the caudal ACC ($d_{\text{left hemisphere}}$ =-0.261, $d_{\text{right hemisphere}}$ = -0.324), left rostral ACC (d = -0.248), lateral OFC ($d_{\text{left hemisphere}}$ = -0.364, $d_{\text{right hemisphere}}$ = -0.298), caudal dlPFC ($d_{\text{left hemisphere}}$ =0.310, $d_{\text{right hemisphere}}$ = -0.259), rostral dlPFC ($d_{\text{left hemisphere}}$ =-0.480, $d_{\text{right hemisphere}}$ = -0.221), superior dlPFC ($d_{\text{left hemisphere}}$ =-0.272, $d_{\text{right hemisphere}}$ = -0.634) and left pars opercularis (*d* = -0.251), pars orbitalis ($d_{\text{left hemisphere}}$ =-0.671, $d_{\text{right hemisphere}}$ = -0.477) and pars triangularis ($d_{\text{left hemisphere}}$ =-0.609, $d_{\text{right hemisphere}}$ = -0.341). These effects reflect an increase in oscillatory power in

these regions among girls with LOC-eating, and a decrease or lesser increase in oscillatory power in these regions among girls with no LOC-eating. The only exceptions to this pattern were observed in the right lateral OFC, where girls with LOC-eating had a decrease in oscillatory power, albeit a smaller decrease than girls without LOC-eating; and the left caudal dlPFC where girls without LOC-eating had greater increase in oscillatory power than girls with LOC-eating.

AR group. Girls in the AR group without LOC-eating had an increase in the left caudate (EMM[95%CI] = 0.047[0.007 - 0.087], left putamen (EMM[95%CI] = 0.051[0.006 - 0.096]), lateral OFC ($EMM[95\%CI]_{left\ hemisphere}$ = 0.076 [0.033 - 0.118]; $EMM[95\%CI]_{right\ hemisphere}$ = 0.045 [0.003 - 0.087]) , left pars opercularis (EMM[95%CI] = 0.065 [0.025 - 0.106]), pars orbitalis ($EMM[95\%CI]_{left\ hemisphere}$ = 0.064 [0.020 - 0.108]; $EMM[95\%CI]_{right\ hemisphere}$ = 0.075 [0.031 - 0.119]) and left pars triangularis (EMM[95%CI] = 0.068[0.021 - 0.114]). Girls in the AR group with LOC-eating experienced minimal to no change in oscillatory power in any ROI (95% CI for all ROIs contained zero). Within the AR group, there was small to large effects of LOC-eating on the left caudate (d = 0.434), left caudal ACC (d = 0.241), rostral ACC ($d_{left\ hemisphere}$ =0.403, $d_{right\ hemisphere}$ = 0.757), lateral OFC ($d_{left\ hemisphere}$ =0.761, $d_{right\ hemisphere}$ = 0.893), medial OFC ($d_{left\ hemisphere}$ =0.259, $d_{right\ hemisphere}$ = 0.607), caudal dlPFC ($d_{left\ hemisphere}$ = -0.296, $d_{right\ hemisphere}$ = -0.218), right rostral dlPFC (d = 0.351), superior dlPFC ($d_{left\ hemisphere}$ =-0.212, $d_{right\ hemisphere}$ = -0.261), right pars opercularis (d = -0.290), pars orbitalis ($d_{left\ hemisphere}$ = 0.733, $d_{right\ hemisphere}$ = 0.903), and right pars triangularis (d = 0.328). In general, these differences reflect greater decreases or lesser increases in oscillatory power among girls with LOC-eating, and increases in oscillatory power among girls without LOC-eating. The dlPFC was an exception to this pattern, with mixed findings across different regions of the dlPFC. Regarding changes in oscillatory power in the dlPFC, girls with LOC-eating had a greater increase in caudal dlPFC,

decrease in rostral dlPFC, and a greater increase or lesser decrease in the superior dlPFC. Girls without LOC-eating had a smaller increase in the caudal dlPFC, an increase in the rostral dlPFC, and a lesser increase or greater decrease in oscillatory power in the superior dlPFC.

$\Delta_{\text{oscillatory power}}$ during attention deployment (250-500ms)

Control group. Among the control group, there was an increase in oscillatory power in the right caudal ACC (EMM[95%CI] = 0.058 [0.011- 0.105]) and right medial OFC (EMM[95%CI] = 0.041 [0.001- 0.08]). The control group had an increase in oscillatory power in the left pars opercularis (EMM[95%CI] = 0.043 [0.003- 0.082]).

AR group. The AR group had decreases in the left striatum (EMM[95%CI]$_{\text{caudate}}$ =-0.052 [-0.086- -0.017], EMM[95%CI]$_{\text{pallidum}}$ =-0.051 [-0.095- -0.008], EMM[95%CI]$_{\text{putamen}}$ =-0.062 [-0.1- -0.025]), left caudal ACC (EMM[95%CI] = -0.069 [-0.104- -0.033]), left OFC (EMM[95%CI] = -0.062 [-0.102- -0.023]), left rostral dlPFC (EMM[95%CI] = -0.031 [-0.061- -0.002]), and left vlPFC (EMM[95%CI]$_{\text{pars opercularis}}$ =-0.054 [-0.09- -0.017], EMM[95%CI]$_{\text{pars orbitalis}}$ = -0.052 [-0.089- -0.016], EMM[95%CI]$_{\text{pars triangularis}}$ =-0.071 [-0.107- -0.036]) .

Exploratory interaction effects of smartphone program and LOC-eating. The interactive effects of smartphone program and LOC-eating on change in oscillatory power during attention deployment are reported in Supplemental Table 3.

Control group. Among girls in the control group without LOC-eating, oscillatory power had minimal to no change in any ROI (95% CI all contained zero). Girls in the control group with LOC-eating had an increase in the caudal ACC (EMM[95%CI]$_{\text{left hemisphere}}$ = 0.068[0.002 - 0.135]; EMM[95%CI]$_{\text{right hemisphere}}$ =0.099[0.016 - 0.182]), right medial OFC (EMM[95%CI] = 0.070 [<.001 - 0.14]), and right superior dlPFC (EMM[95%CI] = 0.061 [0.005 - 0.117]). Within

the control group, there were small to medium effects of LOC-eating on oscillatory power in the right caudate (d = -0.394), right pallidum (d = -0.414), caudal ACC ($d_{\text{left hemisphere}}$ = -0.729, $d_{\text{right hemisphere}}$ = -0.516), rostral ACC ($d_{\text{left hemisphere}}$ = -0.225, $d_{\text{right hemisphere}}$ = -0.391), lateral OFC ($d_{\text{left hemisphere}}$ = -0.713, $d_{\text{right hemisphere}}$ = 0.251), medial OFC ($d_{\text{left hemisphere}}$ = -0.622 , $d_{\text{right hemisphere}}$ = -0.500), caudal dlPFC ($d_{\text{left hemisphere}}$ = -0.485, $d_{\text{right hemisphere}}$ = -0.262), rostral dlPFC ($d_{\text{left hemisphere}}$ = -0.493, $d_{\text{right hemisphere}}$ = -0.440), superior dlPFC ($d_{\text{left hemisphere}}$ = -0.531, $d_{\text{right hemisphere}}$ = -0.765), pars opercularis($d_{\text{left hemisphere}}$ = -0.370, $d_{\text{right hemisphere}}$ = -0.300), pars orbitalis ($d_{\text{left hemisphere}}$ = -0.447, $d_{\text{right hemisphere}}$ = 0.323), and left pars triangularis (d = -0.544). These effects reflect greater increases in oscillatory power among girls with LOC-eating, and decreases or lesser increases in oscillatory power among girls without LOC-eating. The only exceptions to this pattern were in the right lateral OFC and right pars orbitalis, where girls with LOC-eating had a greater decrease in oscillatory power and girls without LOC-eating had an increase or lesser decrease in oscillatory power.

AR group. Girls in the AR group without LOC-eating had decreased oscillatory power in right rostral ACC (EMM[95%CI] = -0.048[-0.093 - -0.003]), left superior dlPFC (EMM[95%CI] = -0.037[-0.068 - -0.006]), and increased oscillatory power in the right pars triangularis (EMM[95%CI] = 0.053[0.004 - 0.102]). In the left caudal ACC, a decrease in oscillatory power was observed for both girls without LOC-eating (EMM[95%CI] = -0.056[-0.096 - -0.016]) and girls with LOC-eating (EMM[95%CI] = -0.081[-0.158 - -0.004]). Girls in the AR group with LOC-eating also had decreased oscillatory power in the left caudate (EMM[95%CI] = -0.091[-0.166 - -0.016]), left putamen (EMM[95%CI] = -0.107[-0.19 - -0.025]), left lateral OFC (EMM[95%CI] = -0.106[-0.192 - -0.020]), and left vlPFC ($\text{EMM[95\%CI]}_{\text{pars opercularis}}$ = -0.114[-0.194 - -0.034], $\text{EMM[95\%CI]}_{\text{pars orbitalis}}$ = -0.106[-0.185 - -0.026], $\text{EMM[95\%CI]}_{\text{pars triangularis}}$ = -

0.123[-0.201 - -0.045]). Within the AR group, there were small to large effects of LOC-eating on the caudate ($d_{\text{left hemisphere}}$ = 0.688, $d_{\text{right hemisphere}}$ = 0.326), pallidum ($d_{\text{left hemisphere}}$ = 0.391, $d_{\text{right hemisphere}}$ = 0.750), putamen ($d_{\text{left hemisphere}}$ = 0.667, $d_{\text{right hemisphere}}$ = 0.753) left caudal ACC (d = 0.205), right rostral ACC (d = -0.515), lateral OFC ($d_{\text{left hemisphere}}$ = 0.594, $d_{\text{right hemisphere}}$ = 0.211), right medial OFC (d = -0.202), right caudal dlPFC (d = 0.492), rostral dlPFC ($d_{\text{left hemisphere}}$ = 0.474, $d_{\text{right hemisphere}}$ = 0.476) , left superior dlPFC (d = -0.351), pars opercularis ($d_{\text{left hemisphere}}$ = 0.930, $d_{\text{right hemisphere}}$ = 0.860), left pars orbitalis (d = 0.866), and pars triangularis ($d_{\text{left hemisphere}}$ = 0.758, $d_{\text{right hemisphere}}$ = 0.487). These effects reflect a mixed pattern of change across ROIs. Girls with LOC-eating had a decrease in oscillatory power in the striatum, caudal ACC, lateral OFC, dlPFC and vlPFC, wherease girls without LOC-eating had increases or lesser decreases in oscillatory power in these regions. Additionally, girls with LOC-eating had greater increases in oscillatory power in the rostral ACC and medial OFC compared to girls without LOC-eating. Lastly, girls without LOC-eating had a greater decrease in oscillatory power in the left superior dlPFC compared to girls with LOC-eating.

Supplemental Table S1. Unadjusted energy intake prior to and following completion of the smartphone program

	**Total sample (n=48)**		**AR group (n=22)**		**Control group (n=26)**	
	**Pre-program**	**Post-program**	**Pre-program**	**Post-program**	**Pre-program**	**Post-program**
**Total energy intake (kcal)**	975.72, 336.2	1025.05, 394.38	900.31, 353.86	975.78, 432.39	1039.53, 313.18	1066.75, 362.51
**Percentage intake from protein**	14.19, 3.05	14.06, 3.61	14.04, 2.84	14.11, 3.52	14.32, 3.28	14.02, 3.76
**Percentage intake from carbohydrates**	48.75, 7.98	48.14, 8.23	49.69, 7.7	47.32, 9.52	47.96, 8.27	48.83, 7.07
**Percentage intake from fat**	37.06, 5.99	37.8, 5.84	36.28, 5.52	38.57, 6.45	37.72, 6.4	37.15, 5.31

Note. Means and standard deviations presented.

Supplemental Table S2. Interactive Effects of condition and LOC-eating on $\Delta_{\text{oscillatory power}}$ during attention capture (0-250ms following stimulus)

	**Effect of Condition * LOC**	**Control no LOC (*n*=8)**	**Control LOC (*n*=4)**	**Control no LOC - LOC**	**AR no LOC (*n*=11)**	**AR LOC (*n*=3)**	**AR no LOC - LOC**
ROI	β [95% CI]	EMM [95% CI]	EMM [95% CI]	*d*	EMM [95% CI]	EMM [95% CI]	Cohen's *d*
Striatum							
Caudate-lh	-0.134 [-0.288 - 0.019]	<0.001 [-0.046 - 0.047]	0.020 [-0.046 - 0.086]	-0.177	**0.047 [0.007 - 0.087]**	-0.005 [-0.081 - 0.071]	0.434*
Caudate-rh	-0.053 [-0.177 - 0.071]	0.021 [-0.032 - 0.075]	0.043 [-0.033 - 0.119]	-0.175	0.020 [-0.026 - 0.065]	0.027 [-0.06 - 0.114]	-0.054
Pallidum-lh	-0.018 [-0.139 - 0.103]	-0.032 [-0.091 - 0.028]	0.018 [-0.066 - 0.102]	-0.374*	0.049 [-0.002 - 0.099]	0.028 [-0.069 - 0.125]	0.131
Pallidum-rh	-0.035 [-0.179 - 0.109]	0.001 [-0.061 - 0.063]	0.055 [-0.033 - 0.143]	-0.336*	0.015 [-0.038 - 0.068]	0.004 [-0.097 - 0.106]	0.069
Putamen-lh	-0.069 [-0.201 - 0.063]	-0.032 [-0.085 - 0.021]	0.006 [-0.069 - 0.082]	-0.328*	**0.051 [0.006 - 0.096]**	0.041 [-0.046 - 0.128]	0.072
Putamen-rh	-0.002 [-0.157 - 0.152]	-0.002 [-0.059 - 0.055]	0.037 [-0.044 - 0.117]	-0.261*	0.029 [-0.02 - 0.078]	0.012 [-0.082 - 0.105]	0.124
ACC							
Caudal-lh	-0.068 [-0.266 - 0.131]	0.018 [-0.036 - 0.072]	0.052 [-0.024 - 0.128]	-0.261*	-0.025 [-0.071 - 0.022]	-0.058 [-0.146 - 0.031]	0.241*
Caudal-rh	-0.07 [-0.233 - 0.092]	0.034 [-0.018 - 0.086]	0.072 [-0.002 - 0.146]	-0.324*	-0.001 [-0.045 - 0.044]	0.004 [-0.081 - 0.09]	-0.034
Rostral-lh	-0.085	-0.003	0.028	-0.248*	0.040	-0.015	0.403*

	[-0.252 - 0.082]	[-0.055 - 0.049]	[-0.046 - 0.102]		[-0.005 - 0.084]	[-0.101 - 0.07]	
Rostral-rh	-0.071	-0.001	0.006	-0.062	0.020	-0.068	0.757**
	[-0.26 - 0.119]	[-0.047 - 0.045]	[-0.059 - 0.070]		[-0.019 - 0.059]	[-0.143 - 0.006]	
OFC							
Lateral-lh	**-0.197**	-0.032	0.012	-0.364*	**0.076**	-0.024	0.761**
	**[-0.346 - -0.047]**	[-0.082 - 0.018]	[-0.059 - 0.083]		**[0.033 - 0.118]**	[-0.106 - 0.058]	
Lateral-rh	-0.049	-0.047	-0.004	-0.298*	**0.045**	-0.047	0.893***
	[-0.204 - 0.107]	[-0.096 - 0.002]	[-0.074 - 0.065]		**[0.003 - 0.087]**	[-0.127 - 0.034]	
Medial-lh	0.007	-0.012	-0.007	-0.038	0.034	0.003	0.259*
	[-0.163 - 0.177]	[-0.060 - 0.036]	[-0.075 - 0.060]		[-0.007 - 0.074]	[-0.075 - 0.081]	
Medial-rh	-0.014	-0.022	-0.013	-0.076	0.019	-0.041	0.607**
	[-0.168 - 0.14]	[-0.066 - 0.022]	[-0.075 - 0.049]		[-0.019 - 0.057]	[-0.113 - 0.031]	
dlPFC							
Caudal -lh	-0.034	**0.040**	0.012	0.310*	0.004	0.036	-0.296*
	[-0.196 - 0.129]	**[<0.001 - 0.08]**	[-0.044 - 0.069]		[-0.030 - 0.038]	[-0.003 - 0.101]	
Caudal -rh	-0.143	0.016	0.039	-0.259*	0.017	0.046	-0.218*
	[-0.286 - 0.001]	[-0.030 - 0.062]	[-0.027 - 0.104]		[-0.023 - 0.056]	[-0.030 - 0.122]	
Rostral -lh	-0.095	-0.032	0.020	-0.480*	0.034	0.033	0.008
	[-0.246 - 0.055]	[-0.075 - 0.012]	[-0.041 - 0.082]		[-0.003 - 0.071]	[-0.038 - 0.104]	
Rostral -rh	-0.02	0.005	0.024	-0.221*	0.028	-0.011	0.351*
	[-0.178 - 0.137]	[-0.035 - 0.045]	[-0.033 - 0.080]		[-0.006 - 0.062]	[-0.076 - 0.054]	
Superior-lh	0.06	0.011	0.031	-0.272*	-0.022	-0.004	-0.212*
	[-0.104 - 0.223]	[-0.020 - 0.042]	[-0.013 - 0.075]		[-0.048 - 0.005]	[-0.055 - 0.047]	
Superior-rh	-0.064	0.021	**0.060**	-0.634**	0.009	0.03	-0.261*
	[-0.248 - 0.12]	[-0.008 - 0.050]	**[0.101 - 0.019]**		[-0.016 - 0.034]	[-0.018 - 0.078]	
vlPFC							
Pars opercularis-lh	0.021	-0.024	0.005	-0.251*	**0.065**	0.073	-0.066
	[-0.159 - 0.201]	[-0.072 - 0.024]	[-0.063 - 0.072]		**[0.025 - 0.106]**	[-0.004 - 0.151]	
Pars opercularis-rh	-0.072	0.026	0.048	-0.175	0.01	0.052	-0.290*
	[-0.206 - 0.062]	[-0.028 - 0.080]	[-0.029 - 0.124]		[-0.036 - 0.056]	[-0.036 - 0.140]	
Pars orbitalis-lh	**-0.181**	**-0.065**	0.027	-0.671**	**0.064**	-0.026	0.733**
	**[-0.33 - -0.033]**	**[-0.116 - -0.013]**	[-0.046 - 0.100]		**[0.020 - 0.108]**	[-0.110 - 0.058]	
Pars orbitalis-rh	-0.056	-0.051	0.028	-0.609**	**0.075**	-0.043	0.903***

	[-0.223 - 0.112]	[-0.103 - 0.001]	[-0.046 - 0.102]		**[0.031 - 0.119]**	[-0.128 - 0.042]	
Pars triangularis-lh	-0.058	-0.047	0.017	-0.477*	**0.068**	0.061	0.045
	[-0.203 - 0.087]	[-0.102 - 0.008]	[-0.061 - 0.094]		**[0.021 - 0.114]**	[-0.028 - 0.151]	
Pars triangularis-rh	-0.085	0.001	0.042	-0.341*	0.038	-0.007	0.328*
	[-0.24 - 0.069]	[-0.050 - 0.053]	[-0.031 - 0.115]		[-0.006 - 0.082]	[-0.091 - 0.077]	

Note. *small effect Cohen's *d* = .20; **medium effect Cohen's *d* = .50; ***large effect Cohen's *d*= .80. LOC; loss-of-control eating, β; beta, EMM; estimated marginal mean, *d*; Cohen's *d*, AR: attention retraining, -lh; left hemisphere, -rh; right hemisphere, ACC; anterior cingulate cortex, OFC orbitofrontal cortex, dlPFC; dorsolateral prefrontal cortex, vlPFC; ventrolateral prefrontal cortex. Estimated marginal means with a 95% CI that does not contain 0 are in bolded font. Linear mixed models were adjusted for stimuli pairing (HF-NF, LF-NF, HF-LF), age, fat mass (kg) and height (cm) at pre-intervention, race and ethnicity (0 = non-Hispanic White, 1 = other race or ethnicity), and LOC-eating (0 = absent, 1 = present). Estimated marginal mean represents the group-level mean change score (post intervention – pre intervention) of beta band power. A decrease in power corresponds to an increase in activity. Thus, a negative estimated marginal mean reflects increased activity in that brain region post-intervention. A positive estimated marginal mean reflects decreased activity in that brain region post-intervention.

Supplemental Table S3. Interactive Effects of condition and LOC-eating on $\Delta_{oscillatory\ power}$ during attention deployment (250-500ms following stimulus)

	**Effect of Condition * LOC**	**Control no LOC (*n*=8)**	**Control LOC (*n*=4)**	**Control no LOC - LOC**	**AR no LOC (*n*=11)**	**AR LOC (*n*=3)**	**AR no LOC - LOC**
ROI	**β 95% CI**	**EMM [95% CI]**	**EMM [95% CI]**	***d***	**EMM [95% CI]**	**EMM [95% CI]**	**Cohen's *d***
Striatum							
Caudate-lh	-0.096 [-0.249 - 0.056]	0.009 [-0.037 - 0.055]	0.015 [-0.050 - 0.080]	-0.054	-0.012 [-0.051 - 0.027]	**-0.091 [-0.166 - -0.016]**	0.688**
Caudate-rh	-0.078 [-0.227 - 0.071]	0.003 [-0.051 - 0.056]	0.058 [-0.018 - 0.133]	-0.394*	-0.010 [-0.055 - 0.035]	-0.052 [-0.138 - 0.035]	0.326*
Pallidum-lh	-0.032 [-0.173 - 0.11]	-0.016 [-0.074 - 0.043]	-0.006 [-0.088 - 0.077]	-0.074	-0.021 [-0.071 - 0.029]	-0.081 [-0.176 - 0.014]	0.391*
Pallidum-rh	-0.088 [-0.252 - 0.077]	0.007 [-0.051 - 0.065]	0.068 [-0.014 - 0.149]	-0.414*	0.027 [-0.022 - 0.076]	-0.079 [-0.173 - 0.015]	0.750**
Putamen-lh	**-0.155 [-0.294 - -0.017]**	-0.007 [-0.058 - 0.043]	0.008 [-0.063 - 0.080]	-0.140	-0.017 [-0.060 - 0.026]	**-0.107 [-0.19 - -0.025]**	0.667**
Putamen-rh	-0.085 [-0.216 - 0.047]	0.008 [-0.045 - 0.061]	0.029 [-0.046 - 0.104]	-0.165	0.021 [-0.025 - 0.066]	-0.082 [-0.169 - 0.005]	0.753**
ACC							
Caudal-lh	-0.096 [-0.213 - 0.021]	-0.015 [-0.063 - 0.032]	**0.068 [0.002 - 0.135]**	-0.729**	**-0.056 [-0.096 - -0.016]**	**-0.081 [-0.158 - -0.004]**	0.205*
Caudal-rh	-0.096 [-0.29 - 0.097]	0.017 [-0.042 - 0.076]	**0.099 [0.016 - 0.182]**	-0.516**	-0.021 [-0.071 - 0.029]	-0.035 [-0.131 - 0.061]	0.104
Rostral-lh	-0.124 [-0.277 - 0.029]	-0.019 [-0.073 - 0.034]	0.009 [-0.066 - 0.085]	-0.225*	-0.042 [-0.088 - 0.003]	-0.018 [-0.105 - 0.069]	-0.175
Rostral-rh	**-0.161 [-0.302 - -0.02]**	0.009 [-0.044 - 0.061]	0.056 [-0.019 - 0.130]	-0.391*	**-0.048 [-0.093 - -0.003]**	0.024 [-0.062 - 0.110]	-0.515**
OFC							
Lateral-lh	-0.004 [-0.157 - 0.149]	-0.018 [-0.071 - 0.034]	0.06 [-0.014 - 0.135]	-0.713**	-0.019 [-0.064 - 0.026]	**-0.106 [-0.192 - -0.020]**	0.594**

Lateral-rh	0.029<br>[-0.126 - 0.183]	0.026<br>[-0.024 - 0.077]	-0.003<br>[-0.074 - 0.068]	0.251*	0.016<br>[-0.027 - 0.059]	-0.012<br>[-0.094 - 0.071]	0.211*
Medial-lh	-0.106<br>[-0.273 - 0.06]	-0.018<br>[-0.069 - 0.032]	0.054<br>[-0.017 - 0.125]	-0.622**	-0.031<br>[-0.074 - 0.012]	-0.046<br>[-0.128 - 0.036]	0.112
Medial-rh	-0.166<br>[-0.335 - 0.003]	0.012<br>[-0.038 - 0.061]	**0.070**<br>**[<.001 - 0.14]**	-0.500**	-0.022<br>[-0.064 - 0.02]	0.004<br>[-0.076 - 0.085]	-0.202*
dlPFC							
Caudal -lh	**-0.167**<br>**[-0.332 - -0.002]**	-0.009<br>[-0.059 - 0.040]	0.042<br>[-0.028 - 0.113]	-0.485*	0.002<br>[-0.041 - 0.044]	-0.025<br>[-0.106 - 0.056]	0.193
Caudal -rh	-0.019<br>[-0.127 - 0.09]	-0.014<br>[-0.071 - 0.042]	0.023<br>[-0.056 - 0.103]	-0.262*	0.04<br>[-0.008 - 0.088]	-0.028<br>[-0.121 - 0.064]	0.492*
Rostral -lh	-0.086<br>[-0.215 - 0.042]	-0.010<br>[-0.049 - 0.030]	0.039<br>[-0.017 - 0.095]	-0.493*	-0.008<br>[-0.042 - 0.026]	-0.055<br>[-0.120 - 0.010]	0.474*
Rostral -rh	-0.095<br>[-0.248 - 0.058]	-0.010<br>[-0.052 - 0.033]	0.029<br>[-0.031 - 0.089]	-0.440*	0.022<br>[-0.014 - 0.059]	-0.034<br>[-0.103 - 0.035]	0.476*
Superior-lh	0.025<br>[-0.13 - 0.18]	-0.021<br>[-0.057 - 0.016]	0.029<br>[-0.022 - 0.08]	-0.531**	**-0.037**<br>**[-0.068 - -0.006]**	-0.006<br>[-0.065 - 0.053]	-0.351*
Superior-rh	-0.143<br>[-0.335 - 0.049]	-0.015<br>[-0.054 - 0.025]	**0.061**<br>**[0.005 - 0.117]**	-0.765**	0.015<br>[-0.019 - 0.049]	0.005<br>[-0.060 - 0.069]	0.107
vlPFC							
Pars opercularis-lh	**-0.162**<br>**[-0.314 - -0.01]**	0.022<br>[-0.027 - 0.071]	0.064<br>[-0.005 - 0.133]	-0.370*	0.006<br>[-0.035 - 0.048]	**-0.114**<br>**[-0.194 - -0.034]**	0.930***
Pars opercularis-rh	-0.07<br>[-0.251 - 0.111]	-0.014<br>[-0.061 - 0.033]	0.016<br>[-0.051 - 0.082]	-0.300*	0.039<br>[-0.001 - 0.079]	-0.075<br>[-0.152 - 0.002]	0.860***
Pars orbitalis-lh	-0.108<br>[-0.26 - 0.044]	-0.013<br>[-0.062 - 0.035]	0.040<br>[-0.029 - 0.109]	-0.447*	0.001<br>[-0.040 - 0.043]	**-0.106**<br>**[-0.185 - -0.026]**	0.866***
Pars orbitalis-rh	-0.106<br>[-0.276 - 0.064]	-0.011<br>[-0.061 - 0.039]	-0.044<br>[-0.114 - 0.026]	0.323*	0.025<br>[-0.017 - 0.067]	0.021<br>[-0.060 - 0.102]	0.032
Pars triangularis-lh	0.002<br>[-0.15 - 0.153]	-0.002<br>[-0.050 - 0.045]	0.049<br>[-0.018 - 0.117]	-0.544**	-0.020<br>[-0.060 - 0.021]	**-0.123**<br>**[-0.201 - -0.045]**	0.758**
Pars triangularis-rh	-0.095<br>[-0.26 - 0.07]	-0.016<br>[-0.074 - 0.041]	-0.002<br>[-0.083 - 0.079]	-0.129	**0.053**<br>**[0.004 - 0.102]**	-0.028<br>[-0.121 - 0.066]	0.487*

Note. *small effect Cohen's *d* = .20; **medium effect Cohen's *d* = .50; ***large effect Cohen's *d*= .80. LOC; loss-of-control eating, β; beta, EMM; estimated marginal mean, *d*; Cohen's *d*, AR: attention retraining, -lh; left hemisphere, -rh; right hemisphere, ACC; anterior cingulate cortex, OFC orbitofrontal cortex, dlPFC; dorsolateral prefrontal cortex, vlPFC; ventrolateral prefrontal cortex. Estimated marginal means with a 95% CI that does not contain 0 are in bolded font. Linear mixed models were adjusted for stimuli pairing (HF-NF, LF-NF, HF-LF), age, fat mass (kg) and height (cm) at pre-intervention, race and ethnicity (0 = non-Hispanic White, 1 = other race or ethnicity), and LOC-eating (0 = absent, 1 = present). Estimated marginal mean represents the group-level mean change score (post intervention – pre intervention) of beta band power. A decrease in power corresponds to an increase in activity. Thus, a negative estimated marginal mean reflects increased activity in that brain region post-intervention. A positive estimated marginal mean reflects decreased activity in that brain region post-intervention.

Supplemental Figure S1. Schematic of the food-cue visual probe task

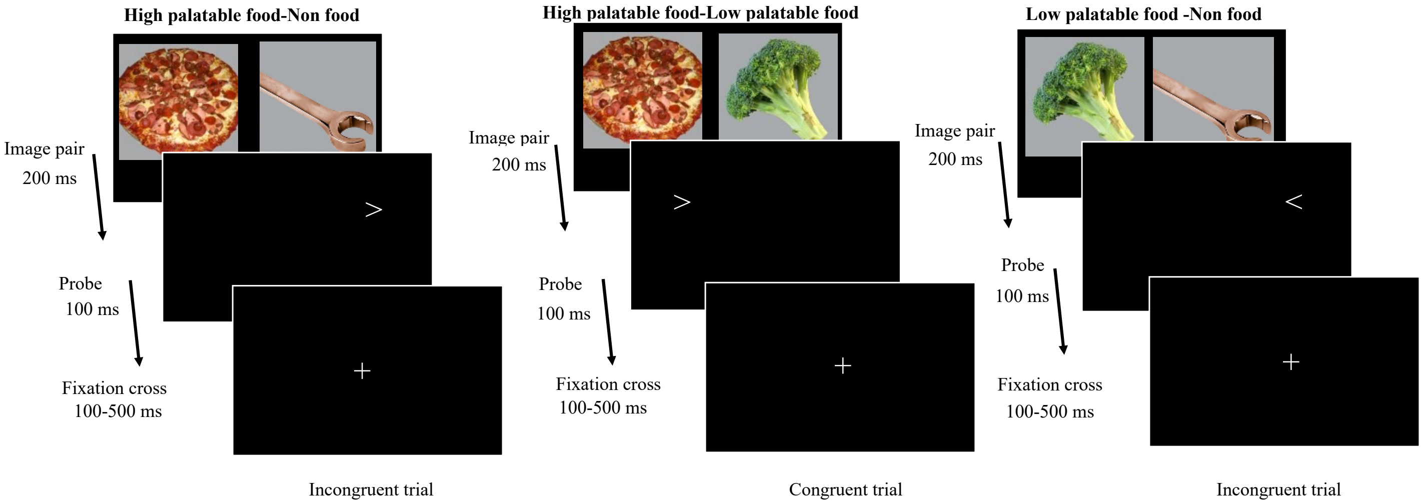

Note. The visual probe task consisted of 180 trials, with 60 trials of each pairing category (e.g., high-palatable food/non-food). During each trial, stimuli from two categories were presented on opposite sides of the screen for 200 ms. Then, both photographs simultaneously disappeared, and a left or right probe (< or >) replaced the spatial position occupied by one of the two stimuli. Participants were told to press the left button for a left pointing arrow or a right button for a right pointing arrow as quickly as possible. The arrows remained on the screen for 100 ms before being replaced by a central fixation cross that was presented for 100, 150, or 500 ms. Trials where the probe appeared behind the more salient cue were considered congruent trials. Trials where the probe appeared behind the less salient cue were considered incongruent trials. The spatial location of each stimulus, and the location of the arrow were counterbalanced across the task and the image pairs were presented in a randomized order.
